# Hepatic PPARα Is Destabilized by SIRT1 Deacetylase in Undernourished Male Mice

**DOI:** 10.3389/fnut.2022.831879

**Published:** 2022-03-28

**Authors:** Ji Ho Suh, Kang Ho Kim, Margaret E. Conner, David D. Moore, Geoffrey A. Preidis

**Affiliations:** ^1^Division of Gastroenterology, Hepatology and Nutrition, Department of Pediatrics, Baylor College of Medicine and Texas Children's Hospital, Houston, TX, United States; ^2^Department of Anesthesiology, McGovern Medical School, The University of Texas Health Science Center at Houston, Houston, TX, United States; ^3^Department of Molecular Virology and Microbiology, Department of Education, Innovation and Technology, Baylor College of Medicine, Houston, TX, United States; ^4^Department of Nutritional Sciences and Toxicology, University of California, Berkeley, Berkeley, CA, United States

**Keywords:** undernutrition, PPARα (peroxisome proliferator-activated receptor alpha), sirtuin-1 (SIRT1), post-translational modification of proteins, mouse models, sex differences

## Abstract

The nutrient sensing nuclear receptor peroxisome proliferator-activated receptor-α (PPARα) regulates the host response to short-term fasting by inducing hepatic transcriptional programming of ketogenesis, fatty acid oxidation and transport, and autophagy. This adaptation is ineffective in chronically undernourished individuals, among whom dyslipidemia and hepatic steatosis are common. We recently reported that hepatic PPARα protein is profoundly depleted in male mice undernourished by a low-protein, low-fat diet. Here, we identify PPARα as a deacetylation target of the NAD-dependent deacetylase sirtuin-1 (SIRT1) and link this to the decrease in PPARα protein levels in undernourished liver. Livers from undernourished male mice expressed high levels of SIRT1, with decreased PPARα acetylation and strongly decreased hepatic PPARα protein. In cultured hepatocytes, PPARα protein levels were decreased by transiently transfecting constitutively active SIRT1 or by treating cells with the potent SIRT1 activator resveratrol, while silencing SIRT1 increased PPARα protein levels. SIRT1 expression is correlated with increased PPARα ubiquitination, suggesting that protein loss is due to proteasomal degradation. In accord with these findings, the dramatic loss of hepatic PPARα in undernourished male mice was completely restored by treating mice with the proteasome inhibitor bortezomib. Similarly, treating undernourished mice with the SIRT1 inhibitor selisistat/EX-527 completely restored hepatic PPARα protein. These data suggest that induction of SIRT1 in undernutrition results in hepatic PPARα deacetylation, ubiquitination, and degradation, highlighting a new mechanism that mediates the liver's failed adaptive metabolic responses in chronic undernutrition.

## Introduction

The peroxisome proliferator-activated receptor (PPAR) family of lipid sensing transcription factors regulates whole-body metabolism and energy balance. The dominant PPAR isoform in human and mouse liver is PPARα, which promotes the adaptive response to short-term fasting by regulating the expression of genes that facilitate ketogenesis, fatty acid oxidation and transport, and nutrient reclamation *via* autophagy (1, 2). However, in chronic undernutrition this adaptation is ineffective, and metabolic abnormalities including dyslipidemia and hepatic steatosis occur (3, 4). Steatosis, decreased hepatic peroxisome abundance, and impaired fatty acid oxidation are observed in rats chronically undernourished by a low-protein diet; these abnormalities are partially ameliorated by a PPARα agonist, suggesting a lack of PPARα signaling in chronic undernutrition (5). We recently confirmed that hepatic PPARα protein levels are dramatically reduced in chronically undernourished mice fed a low-protein, low-fat diet (LPLFD) (6). Mechanisms by which chronic undernutrition decreases hepatic PPARα protein levels have not been defined.

PPARα expression is thought to be regulated primarily at the mRNA level. Consistent with impaired hepatic fatty acid oxidation in non-alcoholic steatohepatitis (NASH), mRNA expression of *PPARA* is decreased in liver biopsies obtained from patients with NASH and inversely correlates with the severity of steatosis (7). Similarly, livers from patients infected with hepatitis C virus reveal profoundly decreased expression of PPARα at the transcriptional and protein levels (8–11). Mechanisms of transcriptional repression of PPARα by interleukin-6 (12), interleukin-1β (13), and tumor necrosis factor-α (14), as well as silencing by numerous micro-RNAs (15–18) have been described. In contrast, post-translational regulation of PPARα protein stability is poorly understood.

This study aimed to identify mechanisms underlying the dramatic loss of hepatic PPARα protein in undernutrition. Our data reveal that undernutrition induces the expression of sirtuin-1 (SIRT1), a NAD-dependent deacetylase that is known to decrease levels of other nuclear receptors including PPARγ by proteasome-mediated degradation. We identify PPARα as a novel target for SIRT1-mediated deacetylation, ubiquitination, and proteasomal degradation, and demonstrate that hepatic PPARα protein levels can be rescued in undernourished mice by inhibiting SIRT1. These results suggest SIRT1 inhibition as a potential therapy for undernutrition-induced liver and metabolic dysfunction.

## Materials and Methods

### Animal Studies

Wild-type C57BL/6J mice (Charles River Laboratories, Wilmington, MA) were housed in the Baylor College of Medicine Center for Comparative Medicine in a temperature-controlled 14:10-h light–dark room. Dams with 8-day-old pups were randomized to receive a purified LPLFD (5% fat, 7% protein, and 88% carbohydrate) or an isocaloric control diet (15% fat, 20% protein, and 65% carbohydrate; #D09081701B and #D09051102, Research Diets, New Brunswick, NJ) *ad libitum* to model undernutrition (19). On day-of-life 21, pups were weaned to their respective dams' diets and continued on the LPLFD or control diet *ad libitum* for the remainder of the experiment.

Hepatic PPARα was manipulated in 8 week old male mice maintained on either diet by giving intraperitoneal injections of the SIRT1 inhibitor selisistat/EX-527 (#E7034, Sigma-Aldrich, St. Louis, MO), 10 mg/kg/day for 3 consecutive days. Alternatively, mice received a single intraperitoneal injection of 1 mg/kg of the proteasome inhibitor bortezomib (#2204, Cell Signaling Technology, Danvers, MA). Control groups for both experiments received equivalent volumes of sterile PBS. After 24 h, mice were euthanized by CO_2_ inhalation. Liver lobes were harvested and stored at −80°C prior to analysis. All animal experiments were conducted in accordance with the Baylor College of Medicine Institutional Animal Care and Use Committee guidelines.

### Cell Culture and Transient Transfection

HepG2 cells (American Type Culture Collection, Manassas, VA) were maintained in Dulbecco's modified Eagle's medium supplemented with 10% fetal bovine serum (Thermo Fisher Scientific, Waltham, MA) in 12- or 24-well-plates. Cells were transfected with expression vectors for wild type or H363Y mutant (dominant negative) human SIRT1 (#1791 and 1792, Addgene, Waltham, MA) (20) or an equivalent amount of empty expression vector, using FuGENE HD Transfection Reagent (Promega, Madison, WI) according to the manufacturer's protocol. Alternatively, cells were transfected with 100 nM SIRT1 siRNA or control siRNA (#12241S and 6568S, Cell Signaling Technology, Danvers, MA) using Lipofectamine RNAiMAX Transfection Reagent (Invitrogen, Waltham, MA) according to the manufacturer's instructions. Cells were harvested 24–36 h following transfection. Alternatively, 100 μM resveratrol (#1418, Tocris, Bristol, UK) or an equivalent volume of sterile water was added to cultures, and cells were harvested 24 h later.

### Western Blot

Liver tissue was homogenized in RIPA buffer. Lysate (20–50 μg) was loaded onto NuPAGE 4–12% Bis-Tris precast gels and transferred onto PVDF Transfer Membranes (Thermo Fisher Scientific, Waltham, MA). Primary antibodies to PPARα (#PA5-85125, Thermo Fisher Scientific, Waltham, MA), SIRT1 (#2028, Cell Signaling Technology, Danvers, MA), PPARγ (#16643-1-AP, Thermo Fisher Scientific, Waltham, MA), or the proteasome marker PSMB5 (# PA1-977, Thermo Fisher Scientific, Thermo Fisher Scientific, Waltham, MA) were applied. For HepG2 cell experiments, primary antibodies to PPARα (#MA1-822, Thermo Fisher Scientific, Waltham, MA) and SIRT1 #9475 (Cell Signaling Technology, Danvers, MA) were used. GAPDH (#14C10, Cell Signaling Technology, Danvers, MA) served as a housekeeping protein for all western blots. Horseradish peroxidase-conjugated secondary antibodies were applied and protein was visualized with Chemiluminescent Substrate (Thermo Fisher Scientific, Waltham, MA) on the Amersham Imager 600 (GE Healthcare Life Sciences, Marlborough, MA).

### Acetylation and Ubiquitination Assays

Immunoprecipitation was performed by incubating 400 μg liver or cell lysate with 2 μg anti-PPARα antibody (#MA1-822, Thermo Fisher Scientific, Waltham, MA) or anti-PPARγ antibody (#MA5-14889, Thermo Fisher Scientific, Waltham, MA) and Protein A/G beads (Santa Cruz Biotechnology, Santa Cruz, CA) for 12 h at 4°C. Antibody-conjugated beads were washed three times with RIPA buffer at 4°C, then PPAR protein was eluted in protein loading buffer and analyzed by SDS-PAGE. Western blot analysis was performed using antibodies to acetylated lysine (#9441, Cell Signaling Technology, Danvers, MA) or ubiquitin (#58395, Cell Signaling Technology, Danvers, MA).

### Real-Time Quantitative Polymerase Chain Reaction

Total RNA was isolated from 30 to 50 mg liver or cultured hepatocytes using TRIzol Reagent (Invitrogen, Carlsbad, CA) and quantified with a NanoDrop 2000c spectrophotometer (Thermo Fisher Scientific, Waltham, MA). Complementary DNA was synthesized from 1 μg RNA using amfiRivert cDNA Synthesis Platinum Master Mix (GenDEPOT, Inc., Katy, TX). SYBR Green PCR Master Mix (Thermo Fisher Scientific, Waltham, MA) was used on the StepOnePlus Real-Time PCR System (Applied Biosystems, Foster City, CA). Relative expression level to GAPDH was calculated by the comparative cycle threshold (ΔΔCt) method. The following primer sequences were used:

Mouse SIRT1 F-5′-CGGCTACCGAGGTCCATATAC-3′ R-5′-CAGCTCAGGTGGAGGAATTGT-3′

Mouse PPARα F-5′-ACAAGGCCTCAGGGTACCA-3′ R-5′-GCCGAAAGAAGCCCTTACAG-3′

Human SIRT1 F-5′-TGGCAAAGGAGCAGATTAGTAGG-3′ R-5′-CTGCCACAAGAACTAGAGGATAAGA-3′

Human PPARα F-5′-CTATCATTTGCTGTGGAGATCG-3′ R-5′-AAGATATCGTCCGGGTGGTT-3′

### Statistics

All data were normally distributed, thus results are presented as mean ± SD. For experiments consisting of two groups, the 2-tailed student *t*-test was used. For experiments involving more than 2 treatment groups, 1-way analysis of variance (ANOVA) was performed, and when the global test was significant (*P* < 0.05), *post-hoc* Sidak's multiple comparisons test was used to determine between-group differences. Calculations were made using Prism 9.2.0 (GraphPad Software, San Diego, CA).

## Results

### Undernourished Male Mice Have Reduced Hepatic PPARα Protein Levels Despite Increased PPARα Transcript

**Figure 1:**
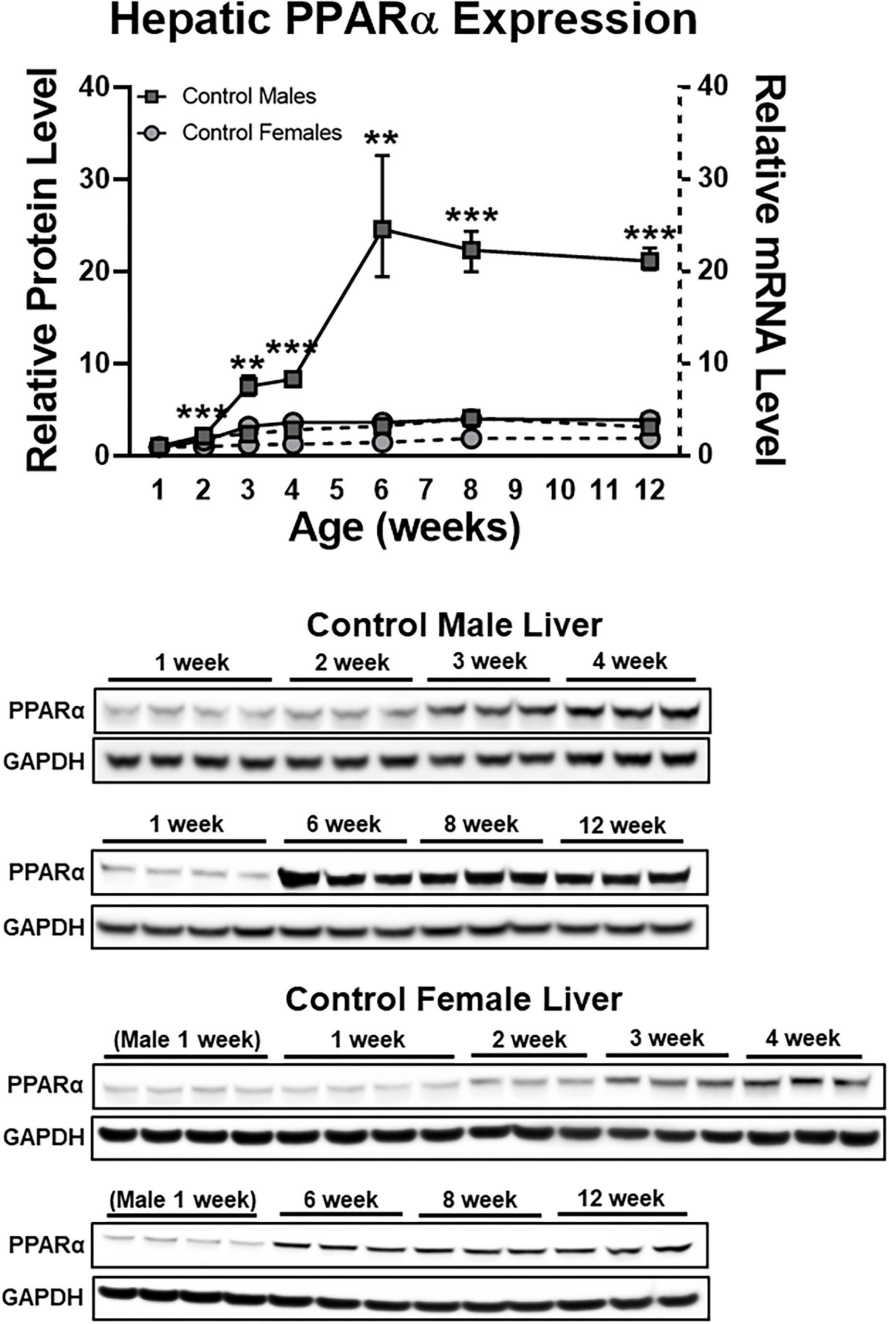
Early life sex differences in hepatic PPARα expression at the protein (solid lines) and mRNA (dashed lines) levels. Although sex differences were most striking with respect to protein expression, mRNA expression also was greater in males compared to females (*P* < 0.05) at every time point after 1 week. *N* = 3–4 mice per time point per group; bars denote range. All data were normalized to 1 week old males. Mean + range; *N* = 3–4; ***P < 0.001; **P < 0.01 between sexes at a given time point for protein expression. GAPDH, glyceraldehyde 3-phosphate dehydrogenase; PPARα, peroxisome proliferator-activated receptor-α.

**Figure 2:**
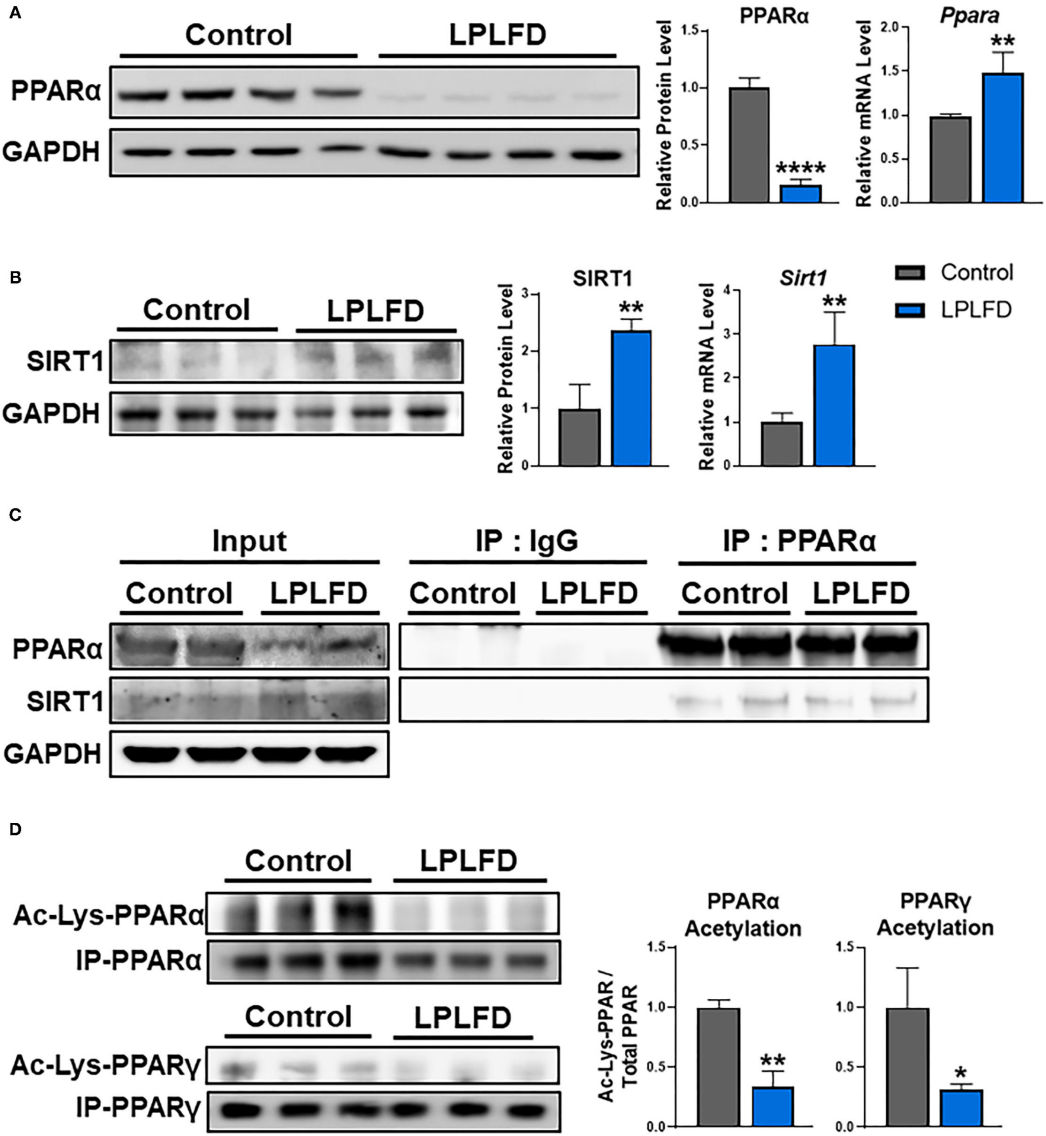
Effects of undernutrition on hepatic PPARα expression and acetylation in male mice. **(A)** Whole livers from undernourished mice contained decreased PPARα protein and increased PPARα transcript relative to livers from control male mice. **(B)** Expression of SIRT1 was increased in undernourished livers at both the protein and the transcriptional level. **(C)** SIRT1 protein was detected bound to PPARα purified from whole liver by immunoprecipitation, suggesting a direct interaction in control and undernourished mouse livers. **(D)** PPAR protein was immunoprecipitated from liver, then comparable amounts were analyzed by western blot to illustrate decreased acetylation of both PPARα, which is not known to be an acetylation target of SIRT1, and PPARγ, which is a well-known acetylation target of SIRT1. Mean + SD; *n* = 2–4; ****P < 0.0001; **P < 0.01; *P < 0.05. Ac-Lys, acetylated lycine; GAPDH, glyceraldehyde 3-phosphate dehydrogenase; IgG, immunoglobulin G; IP, immunoprecipitation; LPLFD, low-protein, low-fat diet; PPAR, peroxisome proliferator-activated receptor; SIRT1, NAD-dependent deacetylase sirtuin-1.

We recently reported that undernutrition dramatically decreases hepatic concentrations of the nuclear receptor PPARα in male mice (6). This effect is not observed in female mice, which have low hepatic PPARα protein expression at baseline (Figure 1). In this study, we sought to identify mechanisms to account for this ~85% decrease in hepatic PPARα protein (*P* < 0.0001; Figure 2A) in undernourished males. In contrast to expectations that PPARα levels are regulated primarily at the transcriptional level (21), qPCR showed a 1.5-fold compensatory increase in *Ppara* transcript in undernourished livers (*P* = 0.006; Figure 2A), leading us to consider post-transcriptional mechanisms of PPARα protein loss.

### Induction of SIRT1 Corresponds With PPARα Deacetylation in Undernourished Livers

SIRT1 binds to and deacetylates multiple members of the nuclear receptor family of transcription factors. Deacetylation of PPARγ (22), farnesoid-X-receptor (23), liver X receptor-α (24), or thyroid receptor-β1 (25) in response to SIRT1 overexpression results in reduced levels of deacetylated nuclear receptors *via* ubiquitin-mediated proteasomal degradation. Although SIRT1 is known to interact directly with PPARα (26), whether this interaction results in PPARα deacetylation and degradation, and also whether SIRT1 is overexpressed in chronic undernutrition, was unknown. We used western blot and qPCR to determine expression levels of SIRT1 in liver from control and undernourished male mice. Livers from undernourished mice showed 2.4-fold increased expression of SIRT1 protein (*P* = 0.007) and 2.8-fold increased expression of *Sirt1* transcript (*P* = 0.004; Figure 2B). Next, we used co-immunoprecipitation to confirm that PPARα and SIRT1 directly interact in undernourished mouse livers (Figure 2C). These findings led us to explore whether increased hepatic expression of SIRT1 in undernutrition is a potential cause of PPARα protein degradation.

To determine whether PPARα deacetylation mechanistically links increased SIRT1 expression with decreased PPARα protein in the chronically undernourished liver, we purified PPARα by immunoprecipitation, then quantified the relative amount of acetylation using comparable amounts of total immunoprecipitated PPARα from control and undernourished livers by western blot. In parallel, we examined acetylation of PPARγ, which is a widely known SIRT1 deacetylation target (22). In accord with their increased expression of SIRT1, undernourished livers contained markedly decreased acetylation of the immunoprecipitated PPARα and PPARγ relative to control livers (Figure 2D). Taken together, these data suggest that PPARα may be a direct target for deacetylation by SIRT1.

### SIRT1 Negatively Regulates PPARα Protein Levels

**Figure 3:**
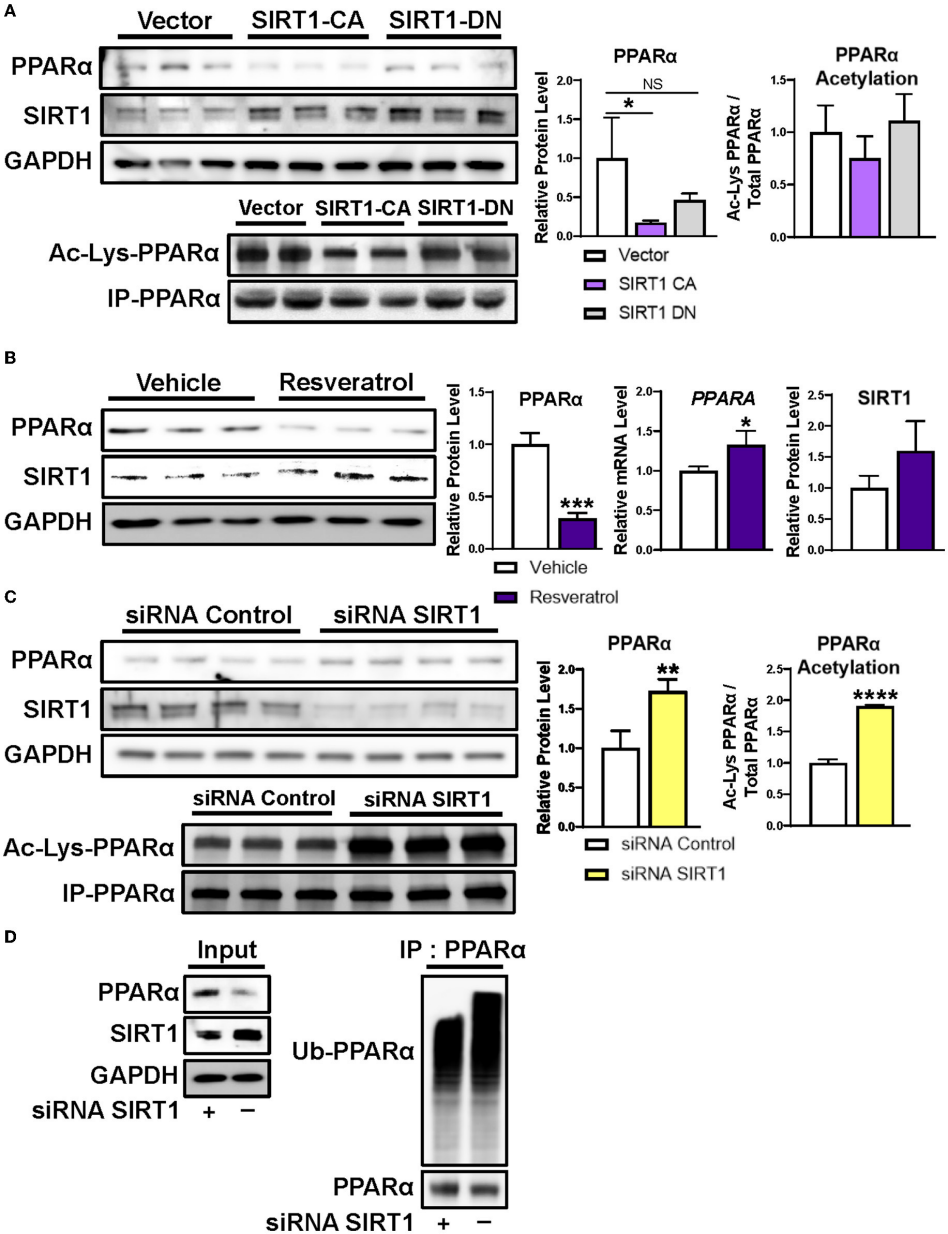
Regulation of PPARα protein level by SIRT1. **(A)** Transient transfection of human-derived HepG2 hepatocellular carcinoma cells with constitutively active SIRT1 decreased PPARα protein levels and acetylation, whereas transfection with dominant negative SIRT1 did not. **(B)** Incubating HepG2 cells with 100 μM of the natural SIRT1 activator resveratrol decreased PPARα protein and increased PPARα mRNA, similar to our mouse model of undernutrition. **(C)** Silencing SIRT1 in HepG2 cells increased PPARα protein and acetylation and **(D)** decreased ubiquitination of PPARα. Mean + SD; *n* = 3–4; ****P < 0.0001; ***P < 0.001; **P < 0.01; *adjusted *P* < 0.05. CA, constitutively active; DN, dominant negative; GAPDH, glyceraldehyde 3-phosphate dehydrogenase; IP, immunoprecipitation; NS, not significant; PPARα, peroxisome proliferator-activated receptor-α; siRNA, small interfering RNA; SIRT1, NAD-dependent deacetylase sirtuin-1; Ub-PPARα, ubiquitinated PPARα.

Next, we sought to determine whether altering SIRT1 expression influences PPARα protein levels. First, we overexpressed constitutively active or dominant negative forms of SIRT1 in HepG2 human hepatoma cells. Indeed, overexpression of constitutively active SIRT1, but not of a dominant negative SIRT1, decreased PPARα protein and acetylation (Figure 3A). Second, we treated HepG2 cells with the natural SIRT1 activator resveratrol (27), and confirmed decreased PPARα protein in conjunction with increased *PPARA* transcript (Figure 3B), in accord with our mouse model of undernutrition. Third, we transfected HepG2 cells with SIRT1 siRNA or non-silencing siRNA control and found that loss of SIRT1 results in increased PPARα protein and acetylation (Figure 3C). These data indicate that SIRT1 negatively regulates PPARα protein levels through deacetylation.

SIRT1 deacetylation causes ubiquitination and subsequent proteasome mediated degradation of multiple nuclear receptors (22–25). To determine whether SIRT1 deacetylation also results in ubiquitination and degradation of PPARα, we assessed ubiquitination of PPARα immunoprecipitated from HepG2 cells after treatment with the siRNAs in the above experiment. As expected, silencing SIRT1 decreased ubiquitination of PPARα and increased its protein level (Figure 3D). All together, these data suggest that hepatic PPARα is reduced in undernourished males due in part to SIRT1 mediated deacetylation followed by ubiquitination and proteasome degradation.

### Hepatic PPARα Is Rescued in Undernourished Mice by Inhibiting SIRT1-Directed Proteasomal Degradation

**Figure 4:**
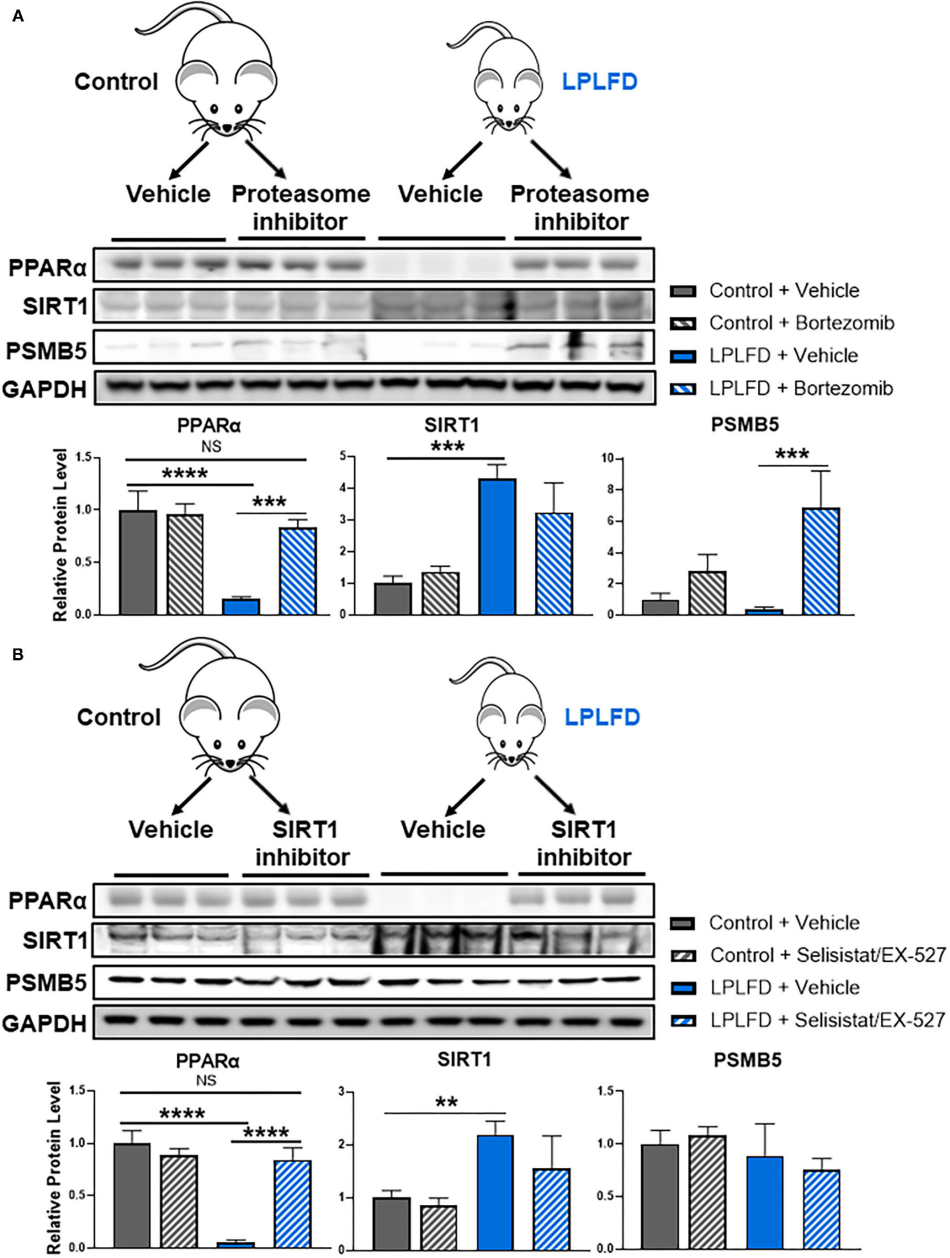
Rescue of hepatic PPARα by blocking SIRT1-mediated proteasomal degradation in undernourished mice. **(A)** Eight-week-old control and undernourished male mice were treated with a single intraperitoneal injection of 1 mg/kg of the proteasome inhibitor bortezomib, or an equivalent volume of sterile PBS, and after 24 hours whole livers were harvested for western blot. Hepatic PPARα levels were rescued completely in undernourished mice treated with proteasome inhibitor. **(B)** Alternatively, control and undernourished mice were treated with three daily intraperitoneal injections of 10 mg/kg/day of the SIRT1 inhibitor selisistat/EX-527, or an equivalent volume of sterile PBS, and 24 h after the third dose, whole livers were harvested for western blot. Hepatic PPARα levels were rescued completely in undernourished mice treated with SIRT1 inhibitor. Mean + SD, *N* = 3, ****adjusted *P* < 0.0001, ***adjusted *P* < 0.001, **adjusted *P* < 0.01. GAPDH, glyceraldehyde 3-phosphate dehydrogenase; LPLFD, low-protein, low-fat diet; NS, not significant; PPARα, peroxisome proliferator-activated receptor-α; PSMB5, proteasome subunit β type 5; SIRT1, NAD-dependent deacetylase sirtuin-1.

Finally, we sought to test the hypothesis that inhibiting SIRT1-directed proteasome degradation would prevent loss of hepatic PPARα in undernutrition. We first administered a single intraperitoneal injection of the proteasome inhibitor bortezomib, or vehicle, to healthy and undernourished male mice, then harvested livers 24 h later for western blot. PPARα was profoundly reduced in livers from vehicle-treated undernourished mice, confirming our previous findings (6). Strikingly, hepatic PPARα levels in undernourished mice were rescued completely by bortezomib (Figure 4A), confirming that proteasome mediated degradation is responsible for loss of hepatic PPARα in undernutrition. In a separate experiment, we administered the potent and specific SIRT1 inhibitor selisistat/EX-527 *via* intraperitoneal injection for 3 consecutive days. Once again, hepatic PPARα levels in undernourished mice were rescued completely by SIRT1 inhibition (Figure 4B). All together, these studies suggest that undernutrition induces expression of SIRT1, which deacetylates PPARα, resulting in subsequent ubiquitination and proteasomal degradation.

## Discussion

The purpose of this study was to identify mechanisms contributing to the loss of hepatic PPARα protein in undernutrition. We employed a mouse model that reproduces key features of chronic undernutrition in humans, including decreased peroxisome abundance and impaired synthesis of bile acids and coagulation factors (6). After ruling out the possibility of transcriptional repression of PPARα, we found that SIRT1 expression was markedly increased in livers from undernourished mice, and we revealed in cultured hepatocytes that SIRT1 negatively regulates PPARα protein levels *via* deacetylation and subsequent ubiquitination and proteasomal degradation. To confirm that this mechanism contributes to hepatic PPARα protein loss in undernutrition, we treated undernourished mice with a proteasome inhibitor or with a SIRT1 inhibitor and found that either drug fully restored hepatic PPARα protein levels.

SIRT1 negatively regulates levels of multiple proteins within the nuclear receptor family *via* deacetylation and subsequent ubiquitin-proteasomal degradation. Previous co-immunoprecipitation studies revealed that SIRT1 physically interacts with PPARα within nuclear protein complexes, but whether this interaction influences PPARα acetylation and protein levels was not known (26). This study confirmed direct interaction between PPARα and SIRT1 in undernourished mouse livers, and is the first study to our knowledge to identify PPARα as a SIRT1 deacetylation and degradation target. Ongoing work in sirtuin-targeted drug discovery (28) may ultimately lead to the consideration of SIRT1 inhibitors as therapeutic alternatives to or amplifiers of PPARα agonists (e.g., fibrates) for metabolic disorders.

For some nuclear receptors SIRT1 serves as a coactivator, increasing the expression of positive transcriptional targets of the receptor prior to its degradation (22–25). It has been proposed that deacetylation might facilitate the release of corepressors or the recruitment of coactivators, or enhance clearing from the promoter for subsequent rounds of transcription (24). Congruent with this working model, transcriptional analysis of our undernourished mouse livers revealed marked upregulation in PPAR signaling, including induction of the positive PPARα target genes *Fgf21, Acot1*, and *Cyp4a14* (6). PPARα is strongly activated in the fasted state (2), and it remains to be determined whether similarly strong PPARα activation is detected during the initial phases of acute undernutrition.

Acetylation sites have been described on PPARγ, including the lysine (K) residues K184/185, K268, and K293, which are confirmed to be SIRT1 deacetylation targets (22, 29, 30). We are not aware of any analogous acetylation sites that have been identified for PPARα. To begin to address this knowledge gap, we probed the PPARα gene sequence of seven mammalian species with a K-acetyltransferase predictive algorithm (31). We identified K232 as a highly conserved residue that is most likely to be responsible for PPARα acetylation (Supplementary Table 1). Further analyses to validate the functional implications of this putative PPARα acetylation site are needed.

SIRT1 induction by calorie restriction is an ancient evolutionary biological stress response that slows aging by increasing the long-term function and survival of cells (32). During periods of fasting that last 12–24 h, SIRT1 deacetylates and activates the PPARγ coactivator PGC-1α to increase fatty acid oxidation and decrease lipogenesis and fat storage (26, 33, 34). SIRT1 similarly deacetylates sterol regulatory element binding protein, farnesoid-X-receptor, and liver-X-receptor to increase bile acid biosynthesis and reduce cholesterol (24, 35). In addition, deacetylated PGC-1α increases the expression of CYP7A1 and CYP8B1 to increase bile acid synthesis from cholesterol (36–38). These pathways serve to protect the host from dyslipidemia, but presumably they require levels of PPARα protein that exceed what is present during chronic undernutrition. We propose that the metabolic benefits of short-term coactivation by SIRT1 of transcriptional targets of PPARα gradually are lost as acute undernutrition progresses to chronic undernutrition, as new PPARα protein synthesis cannot keep up with continuous deacetylation, ubiquitination, and proteasome degradation. In this model, loss of PPARα in response to chronically increased SIRT1 expression undermines the beneficial impact of decreased acetylation of PGC-1α and potentially other SIRT1 targets.

We explored this mechanism of post-translational PPARα protein degradation exclusively in male mice because undernutrition does not decrease hepatic PPARα protein in female mice (6). Indeed, sex differences in PPARα protein levels in mice first appear between the second and third weeks of life, and by 6 weeks of life livers from male mice contain ~13-fold as much PPARα protein as livers from female mice (Figure 1). In our model, undernutrition was initiated during the second week of life; it remains to be determined whether increased SIRT1 expression would cause PPARα deacetylation and degradation if malnourishment was initiated after mice reached adulthood. Sex differences in PPARα expression recently were reported in human monocytes (39). In addition to the known interactions between estrogen and PPARα transcriptional activity (40), sexual dimorphism of PPARα protein levels could have important implications for sex differences in the frequency of metabolic disorders and their treatment (41). Finally, because SIRT1 deacetylates histone proteins as well as non-histone proteins, hepatic SIRT1 induction in undernutrition has the potential to induce epigenetic effects much broader than those uncovered here. Histone deacetylation may be especially important early in development, given the clear link between early-life undernutrition and long-term risk for obesity and metabolic disorders; this link is presumed to have an epigenetic basis (42). Whether acute and long-term metabolic effects of early-life undernutrition could be mitigated by therapeutic strategies that preserve PPARα protein warrants further investigation.

## Data Availability Statement

The raw data supporting the conclusions of this article will be made available by the authors, without undue reservation.

## Ethics Statement

The animal study was reviewed and approved by Baylor College of Medicine Institutional Animal Care and Use Committee.

## Author Contributions

JS, KK, MC, DM, and GP were responsible for the design of the study. JS performed the experiments. JS and GP performed data, statistical analyses, and prepared the manuscript. KK, MC, DM, and GP provided assistance and knowledge that was vital to the completion of the manuscript. All authors contributed to the review of the manuscript.

## Funding

GP received funding from National Institute of Diabetes and Digestive and Kidney Diseases (Grants K08 DK-113114, R03 DK-129495, and P30 DK-056338), which funds the Texas Medical Center Digestive Diseases Center. Publication costs were generously supported by the Texas Children's Hospital Young Investigators Endowed Fund.

## Conflict of Interest

The authors declare that the research was conducted in the absence of any commercial or financial relationships that could be construed as a potential conflict of interest.

## Publisher's Note

All claims expressed in this article are solely those of the authors and do not necessarily represent those of their affiliated organizations, or those of the publisher, the editors and the reviewers. Any product that may be evaluated in this article, or claim that may be made by its manufacturer, is not guaranteed or endorsed by the publisher.
