# Supplementary material for: Hepatic PPARα Is Destabilized by SIRT1 Deacetylase in Undernourished Male Mice
Source: Front Nutr. 2022 Mar 28;9:831879. doi: 10.3389/fnut.2022.831879 (PMC8997242; doi:10.3389/fnut.2022.831879)
Supplement: Supplementary file 1 [file Table_1.DOCX]

**Supplemental Table 1. Predicted PPARα acetylation sites**

| **Species** | **Acc. No.** | **Lys^232^** | **Prediction***  **(P value)** | **Lys^252^** | **Prediction***  **(P value)** |
| --- | --- | --- | --- | --- | --- |
| *Mus musculus*  (house mouse) | NP_035274.2 | KARVILAG**K**TSNNPPFV | 0.0641 | METLCMAE**K**TLVAKMVA | 0.3281 |
| *Rattus norvegicus* (Norway rat) | NP_037328.1 | KARVILAG**K**TSNNPPFV |  | METLCMAE**K**TLVAKMVA |  |
| *Bos Taurus* (cattle) | NP_001029208.1 | KARVILAG**K**TNNNPPFV | 0.0373 | METLCMAE**K**TLVAKLVA | 0.4443 |
| *Equus caballus* (horse) | NP_001229482.1 | KARVILAG**K**ASNHPPFV | 0.0211 | METLCMAE**K**TLVAKLVA |  |
| *Sus scrofa* (pig) | NP_001037991.1 | KARVILAG**K**ASNNPPFV | 0.0635 | METLCMAE**K**TLVAKLVA |  |
| *Macaca mulatta*  (Rhesus monkey) | NP_001028201.1 | KARVILSG**K**ASNNPPFV | 0.0634 | METLCMAE**K**TLVAKLVA |  |
| *Homo sapiens* (human) | NP_005027.2 | KARVILSG**K**ASNNPPFV |  | METLCMAE**K**TLVAKLVA |  |

*Full protein sequence was analyzed by ASEB, a web server for KAT-specific acetylation site prediction.^31^
